# Validation of the new consensus criteria for the diagnosis of corticobasal degeneration

**DOI:** 10.1136/jnnp-2013-307035

**Published:** 2014-02-12

**Authors:** S K Alexander, T Rittman, J H Xuereb, T H Bak, J R Hodges, J B Rowe

**Affiliations:** 1 Addenbrooke's Hospital, Cambridge, UK; 2 Department of Clinical Neurosciences, University of Cambridge, Cambridge, UK; 3 Department of Pathology, University of Cambridge, Cambridge, UK; 4 School of Philosophy, Psychology and Language Sciences, University of Edinburgh, Edinburgh, UK; 5 Neuroscience Research Australia, University of New South Wales, Sydney, New South Wales, Australia

**Keywords:** Alzheimer'S Disease, Dementia, Neuropathology, Corticobasal Degeneration

## Abstract

**Background:**

Corticobasal degeneration (CBD) is a complex neurodegenerative disorder. Accurate diagnosis is increasingly important, with the advent of clinical trials of drugs aimed at modifying the underlying tau pathology. CBD often presents with a ‘corticobasal syndrome’ including impairments of movement and cognition. However, patients with similar corticobasal syndromes can have neurodegenerative pathologies that are not CBD. In addition, patients with CBD may present with aphasia or behavioural change. The clinical diversity of CBD and mimicry by non-CBD pathologies hinders accurate diagnosis.

**Methods:**

We applied the new consensus criteria of Armstrong and colleagues *et al* 1 to a cohort of patients with detailed longitudinal clinical evaluation and neuropathology.

**Results:**

In patients with pathologically confirmed CBD, accuracy of diagnosis was similar under the new and previous criteria: 9/19 (47%) met criteria for probable CBD at presentation, 13/19 (68%) at last clinical assessment. Patients with a corticobasal syndrome but without CBD pathology all (14/14) met the new diagnostic criteria of probable or possible CBD, demonstrating that the new criteria lacks the necessary specificity for an accurate ante mortem clinical diagnosis of CBD. None of the clinical features used in the new criteria were more common in the patients with CBD pathology (n=19) than without (n=14).

**Conclusions:**

The Armstrong criteria usefully broadens the recognised clinical phenotype of CBD but does not sufficiently improve the specificity of diagnosis to increase the power of clinical trials or targeted applications of tau-based disease-modifying therapies. Further work is required to show whether biomarkers could be more effective than clinical signs in the diagnosis of CBD.

## Introduction

Corticobasal degeneration (CBD) is a challenging disease with a complex pattern of neurological impairments and limited diagnostic accuracy, even in specialist clinics.^1–5^ For patients, the outlook is often bleak, with accumulating disability and death, and few treatment options—none of which has been shown to alter the underlying disease course. The importance of CBD lies not only in its relatively young onset, high morbidity and poor prognosis: the pathology also has much in common with other primary tauopathies, including progressive supranuclear palsy (PSP) and frontotemporal lobar degeneration associated with tau pathology (FTLD-tau). This increases the potential impact of novel therapies, but the development of new disease-modifying treatments requires accurate diagnosis. A major limitation has been the similarity of clinical phenotypes between CBD and ‘CBD mimics’ caused by other pathology such as Alzheimer's disease (AD).

Unfortunately, the published literature has been inconsistent in terminology related to CBD. In this article, we use ‘CBD’ to refer only to cases with a neuropathologically confirmed tau pathology affecting glia and neurons in a characteristic distribution.^2^ We use ‘CBD mimic’ to refer to cases of clinically-suspected CBD where the postmortem pathology was not CBD. Clinical diagnostic criteria for CBD (‘clinical CBD’) identify a phenotype, or syndrome, associated with CBD that we call a corticobasal syndrome (CBS). CBS includes a mixed movement disorder (eg, levodopa-unresponsive rigidity associated with apraxia, dystonia, myoclonus and alien limb) and impaired cognition.

In early reports, CBD was principally thought of as a movement disorder that did not compromise higher order cognition.^6^ ^7^ More recently, a broader clinical phenotype of CBD pathology has emerged in several sets of consensus criteria for CBD and CBS^1^ ^5^ ^7^ Cognitive features were first included by Boeve *et al*^7^ with speech apraxia and frontal executive dysfunction. Bak and Hodges later expanded the cognitive profile of CBS (including CBD and non-CBD aetiologies) to include visuospatial impairment, speech and language impairment, with equal weighting of motor and cognitive features.^5^ ^8^

Several diagnostic criteria have been proposed for CBS and CBD, but concordance is low between criteria.^8^ Even with detailed clinical evaluation and the widespread availability of structural and functional imaging, misclassification rates of CBS and CBD are high ante mortem. CBD is defined neuropathologically by the abnormal deposition of aggregated 4-repeat tau protein isoforms in neurons and glial cells, as outlined by Dickson *et al*.^2^ In all, 24%–57% patients with CBD (at autopsy) had been correctly diagnosed in their lifetime.^9–11^ Conversely, many patients who were diagnosed with CBS or clinical CBD had another diagnosis by pathology, most commonly AD and frontotemporal dementia.^4^ ^6^

In clinical practice, neuropathology is rarely available ante mortem. Nonetheless, accurate diagnosis of CBD would critically affect enrolment and power of clinical trials and the applicability of emerging disease-modifying therapies that target tau mechanisms of neurodegeneration. The prevalence of CBD, and its potential for ‘orphan disease’ status in regulatory authority approval for new drugs, further increases the need for accuracy and preferably accuracy early in the course of the illness. Both clinicians and clinical trial investigators therefore require sensitive and specific diagnostic criteria for CBD.

In recently published consensus criteria, Armstrong *et al*^1^ sought to improve the accuracy of CBD diagnosis. A significant development in the Armstrong consensus criteria was the use of pooled neuropathologically-proven cases to determine the clinical phenotype of CBD retrospectively. In doing so, there were two key innovations. First, they proposed categories of probable and possible CBD, indicating the degree of certainty of diagnosis. Second, they broadened the clinical phenotype associated with CBD, by including the clinical categories of frontal behavioural-spatial syndrome (FBS), non-fluent/agrammatic variant of primary progressive aphasia (NAV) and progressive supranuclear palsy syndrome (PSPS). Clinical phenotypes and diagnostic criteria from Armstrong *et al* are shown in table 1. These changes acknowledge the spectrum and overlapping phenotypes of tau-related neurodegenerative diseases.^2–4^ Armstrong *et al* also acknowledged that clinical evidence of memory impairment is common, although this was not included in the criteria.

**Table 1:** Armstrong criteria:^1^ (A) proposed clinical phenotypes or syndromes; (B) proposed diagnostic criteria for CBD; (C) exclusion criteria for both clinical research criteria for probable sporadic CBD and possible CBD

(A) Syndrome	Feature
Probable CBS	Asymmetric presentation of two of: (a) limb rigidity or akinesia, (b) limb dystonia, (c) limb myoclonus plus two of: (d) orobuccal or limb apraxia, (e) cortical sensory deficit, (f) alien limb phenomena (more than simple levitation)
Possible CBS	May be symmetric: one of: (a) limb rigidity or akinesia, (b) limb dystonia, (c) limb myoclonus plus one of: (d) orobuccal or limb apraxia, (e) cortical sensory deficit, (f) alien limb phenomena (more than simple levitation)
Frontal behavioural-spatial syndrome (FBS)	Two of: (a) executive dysfunction, (b) behavioural or personality changes, (c) visuospatial deficits
NAV of primary progressive aphasia	Effortful, agrammatic speech plus at least one of: (a) impaired grammar/sentence comprehension with relatively preserved single word comprehension or (b) groping, distorted speech production (apraxia of speech)
Progressive supranuclear palsy syndrome (PSPS)	Three of: (a) axial or symmetric limb rigidity or akinesia, (b) postural instability or falls, (c) urinary incontinence, (d) behavioural changes, (e) supranuclear vertical gaze palsy or decreased vertical saccade velocity

(B)	Clinical research criteria for probable sporadic CBD	Clinical criteria for possible CBD
Presentation	Insidious onset and gradual progression	Insidious onset and gradual progression
Minimum duration of symptoms, years	1	1
Age at onset, years	≥50	No minimum
Family history (two or more relatives)	Exclusion	Permitted
Permitted phenotypes (see table 4 for criteria)	(1) Probable CBS or (2) FBS or NAV plus at least one CBS feature (a–f)	(1) Possible CBS or (2) FBS or NAV or (3) PSPS plus at least one CBS feature (b–f)
Genetic mutation affecting tau (eg, MAPT)	Exclusion	Permitted

**(C)**		

*Exclusion criteria for both clinical research criteria for probable sporadic CBD and possible CBD*		
Evidence of Lewy body disease, multiple system atrophy, Alzheimer's disease or amyotrophic lateral sclerosis; semantic or logopenic variant primary progressive aphasia; structural lesion suggestive of focal cause; granulin mutation or reduced plasma progranulin levels; TDP-43 or fused in sarcoma (FUS) mutations		

CBD, corticobasal degeneration; CBS, corticobasal syndrome; NAV, non-fluent/agrammatic variant.

We tested the new Armstrong *et al* criteria using patients from a specialist clinical research centre with detailed longitudinal clinical and neuropathological data. We applied the criteria to patients with CBD and then applied the criteria to patients with CBD mimics. This enables an independent assessment of the Armstrong *et al* criteria for CBD, and provides new information about the performance of the new criteria in patients with diseases that mimic CBD. We asked two specific questions: whether the Armstrong criteria specifically identify CBD cases or not, and whether individual clinical features differentiate CBD from CBD mimics.

## Methods

Patients were recruited from regional specialist clinics for Disorders of Movement and Cognition and Early-Onset Dementia at Addenbrooke's Hospital presenting between 1990 and 2013. Only patients with detailed clinical and pathological information were included. The original exclusion criteria were applied (see table 1). Two groups of patients were studied: those with CBD irrespective of ante mortem clinical diagnosis, and those with CBD mimics, diagnosed clinically with CBD or CBS in life but without CBD pathology. Basic demographic data were similar for the two groups, as given in table 2.

**Table 2:** Demographic data for patients with confirmed CBD pathology (‘CBD’) and clinical diagnosis of CBD but negative pathology (‘CBD mimics’); p values for group differences were not significant for any comparison, by χ^2^ or t tests as appropriate

Pathology	‘CBD’	‘CBD mimics’
Number of patients (n)	19	14
M:F ratio	9:10	7:7
Age at presentation (mean±SD)	67 (8)	69 (9)
Duration of disease, diagnosis to death (year; mean±SD)	4 (3)	5 (3)
MMSE at presentation/30 (mean±SD)	15 (8)	21 (5)
ACE-R total at presentation/100 (mean±SD)	51 (25)	65 (13)

CBD, corticobasal degeneration; MMSE, mini mental state examination.

In keeping with UK law on research and retention of human tissue, all patients with mental capacity were appropriately counselled and provided written informed consent for inclusion in research and brain bank donation. Their next of kin or advocate also supported brain donation as part of local procedures. In the absence of mental capacity, next of kin provided a non-binding declaration of intent after consultation and counselling ante mortem, and then provided written informed consent postmortem. Appropriate ethical approval was obtained for this study (Cambridge Research Ethics Committee).

Clinical records were evaluated for the documented presence or absence of the clinical features used in the diagnostic criteria of Armstrong *et al.* In all patients, the disease was of insidious onset over more than a year. For each clinical feature, we documented whether it was present at diagnosis and whether it was ever present during the patient's clinical course. These clinical features are given in table 3. The documented presence or absence of each feature was summed to generate the denominator to calculate percentage frequencies in the same way as Armstrong *et al.* We applied the Armstrong criteria to each of the patients in our cohorts of CBD and CBD mimics. Difference in the frequency of individual clinical features between the patient groups was examined by χ^2^ analysis. Descriptive statistics were performed in Excel with supplemental analysis in ‘R’ software (http://cran.r-project.org/). Neuropathological examination and diagnosis were undertaken without knowledge of the clinical features.

**Table 3:** Frequency of individual clinical features in patients with pathological CBD in our patient cohort compared with the data of Armstrong *et al*^1^

	At presentation n (%)		During entire course n (%)	
Clinical feature	Published	Our data	Published	Our data
Limb rigidity	65/114 (57)	10/17 (59)	153/180 (85)	13/18 (72)
Bradykinesia/clumsy limb	53/111 (48)	10/17 (59)	126/165 (76)	11/18 (61)
Bradykinesia	–	8/16 (50)	–	11/17 (65)
Clumsy limb	–	10/17 (59)	–	10/18 (56)
Postural instability	20/49 (41)	4/16 (25)	73/94 (78)	8/17 (47)
Falls	27/76 (36)	3/14 (21)	83/111 (75)	8/15 (53)
Abnormal gait	30/92 (33)	5/17 (29)	102/140 (73)	13/18 (72)
Axial rigidity	18/67 (27)	4/15 (27)	68/98 (69)	5/16 (31)
Tremor	17/83 (20)	7/15 (47)	50/127 (39)	8/16 (50)
Limb dystonia	18/91 (20)	4/17 (24)	47/123 (38)	6/18 (33)
Myoclonus	14/94 (15)	2/15 (13)	34/128 (28)	5/15 (33)
Cognitive impairment (overall)	59/114 (52)	14/18 (78)	123/175 (70)	17/19 (89)
Objective cognitive impairment	–	14/18 (78)	–	17/19 (89)
Behavioural changes	52/113 (46)	9/17 (53)	82/150 (55)	14/18 (78)
Limb apraxia	46/102 (45)	11/17 (65)	81/142 (57)	14/18 (78)
Aphasia	40/101 (40)	10/19 (53)	80/155 (52)	11/20 (55)
Depression	21/80 (26)	3/16 (19)	42/82 (51)	4/17 (24)
Cortical sensory loss	20/81 (25)	4/17 (24)	29/107 (27)	5/18 (28)
Alien limb	20/90 (22)	5/17 (29)	24/81 (30)	5/18 (28)
Abnormal eye movement	29/88 (33)	7/16 (44)	90/150 (60)	8/16 (50)
Hyperreflexia	17/57 (30)	6/18 (33)	58/116 (50)	6/18 (33)
Speech changes	18/77 (23)	6/15 (40)	59/112 (53)	7/15 (47)
Abnormal eye movement	29/88 (33)	7/16 (44)	90/150 (60)	8/16 (50)

Data given for the presence/absence of each clinical feature at presentation and during the entire course of diagnosed disease. The denominator used was the number of patients with documented presence/absence of each clinical feature. The percentage frequencies of individual clinical features in our cohort and that of Armstrong *et al*^1^ were strongly correlated: r=0.78 at presentation and r=0.58 during entire course of disease across all variables (Pearson correlation).

CBD, corticobasal degeneration.

## Results

### CBD

In all, 19 patients were identified with CBD and comprehensive and detailed clinical records: 10/19 (52%) of these were given a diagnosis of CBD at presentation and 13/19 (68%) during their lifetime. These diagnostic rates are in the upper range of those previously reported.^1^ ^9^ ^12^ ^13^ The frequencies of individual clinical features in pathologically confirmed CBD were compared with published data, as shown in table 3. There was a strong correlation between the frequency of individual clinical features in our cohort and that of Armstrong *et al*: r=0.78 (p=5×10^−5^) at presentation and r=0.58 (p=7×10^−3^) during entire course of disease. Of note, cognitive impairment was common: present in 78% patients at diagnosis and 89% during the entire course, consistent with recently published data.^1^ ^5^

Patients were classified first according to clinical phenotypes (syndromes): ‘probable CBS’, ‘possible CBS’, ‘FBS’, ‘NAV’, ‘PSPS’, and thereafter according to whether they met diagnostic criteria for ‘probable CBD’ or ‘possible CBD’. The diagnostic criteria for CBD incorporate the clinical phenotype for example, FBS or NAV. Hence, a patient with an FBS phenotype and meeting criteria for probable CBD would be classified as probable CBD and included in the ‘FBS phenotype’ number in parentheses. Diagnoses made on this patient group are given in table 4A. The numbers of patients meeting inclusion criteria for each of the individual categories are given in parentheses, demonstrating the overlapping and inclusive nature of the proposed categories.

**Table 4:** (A) Diagnostic classification for patients with pathologically confirmed CBD using criteria proposed by Armstrong *et al.*^1^ Patients were classified first according to clinical syndromes: ‘probable CBS’, ‘possible CBS’, ‘FBS’, ‘NAV’, ‘PSPS’, and thereafter according to whether they met diagnostic criteria for ‘probable CBD’ and/or ‘possible CBD’. The diagnostic criteria for CBD incorporate the clinical phenotype, for example, FBS or NAV, so a patient with one of these phenotypes and also meeting criteria for probable CBD would be classified as probable CBD and included in the FBS/NAV number in parentheses. (B) Diagnoses given to patients at presentation and final diagnosis during their lifetime

	At presentation	During lifetime
(A) Diagnosis	Primary diagnosis	Primary diagnosis
Probable CBD	9	12
Possible CBD	9 (18 including ‘Probable CBD’)	1 (13 including ‘Probable CBD’)
Probable CBS	3	4
Possible CBS	4 (7)	6 (10)
Frontal behavioural-spatial (FBS)	0 (12)	2 (13)
FBS–NAV overlap	0	1
Non-fluent/agrammatic variant (NAV)	0 (11)	3 (12)
PSP phenotype	1 (1)	0 (6)

(B) Diagnosis	At presentation	During lifetime
CBD	10	13
PNFA	2	2
FTD	4	4
PSP	1	0
Other	2 (1 AD, 1 IPD)	0

AD, Alzheimer's disease; CBD, corticobasal degeneration; FTD, frontotemporal dementia; IPD, idiopathic Parkinson's disease; PNFA, progressive non-fluent aphasia; PSP, progressive supranuclear palsy; PSPS, progressive supranuclear palsy syndrome.

The original diagnoses are given in table 4B. As shown, using Armstrong *et al* criteria, 9/19 were diagnosed with probable CBD at presentation and a further nine patients with possible CBD. By death, this had increased to 12 probable and one possible CBD patients. This compares with correct clinical diagnosis of 10 CBD patients at presentation and 13 by death by previous criteria. The Armstrong criteria suggest that patients who do not meet current CBD criteria are more likely to be diagnosed with one of the other phenotypes associated with predominantly tau pathology (FBS, NAV and PSPS). In this cohort, PSP, CBD, progressive non-fluent aphasia or frontotemporal dementia was the clinical diagnosis in 17/19 patients with CBD at presentation and all 19 patients by death: only two cases had Alzheimer's or Parkinson's disease. These data suggest that the new diagnostic criteria do not significantly improve the rate of accurate diagnosis of CBD pathology.

### CBD mimics

We next asked whether the new criteria improved the differential diagnosis of patients in our cohort with non-CBD pathology. We identified 14 patients diagnosed with clinical CBD or CBS but found to have non-CBD pathology at postmortem. Ten had AD pathology, two had FTLD (with negative tau immunocytochemistry) and two had mixed Lewy body and Alzheimer's pathology (one with cortical Lewy bodies, the other with Lewy bodies in the brainstem only; table 5B). We applied the new Armstrong diagnostic criteria to these CBD mimic patients (table 5A,B).

**Table 5:** (A) Diagnostic classification for patients with ‘CBD mimics’, mimicking CBD clinically but with non-CBD pathology, using criteria proposed by Armstrong *et al.*^1^ (B) Postmortem findings in clinical mimics of CBD

(A) Diagnosis	At presentation	During lifetime
Probable CBD	9	10
Possible CBD	5	4
Probable CBS	2	4
Possible CBS	8 (10)	10 (14)
Frontal behavioural-spatial (FBS) phenotype	0 (7)	0
Non-fluent/agrammatic variant	0 (3)	0
Progressive supranuclear palsy phenotype	0 (1)	0

(B)	Postmortem
Alzheimer's disease	10
Frontotemporal dementia (TDP-43)	2
Mixed Alzheimer's disease/Lewy body disease	2

CBD, corticobasal degeneration; CBS, corticobasal syndrome.

In all, 9/14 (64%) were still classified as probable CBD and a further 5/14 (29%) as possible CBD at presentation; All cases were classified with possible or probable CBD at presentation (table 5A) and throughout the disease course. Only one patient changed diagnostic category from possible to probable CBD, so that 10/14 (71%) were classified as probable CBD and 4/14 (29%) as possible CBD before death. This demonstrates that the new criteria do not effectively rule out patients with CBS but non-CBD pathology. Furthermore, all 14 of the non-CBD patients had neurodegenerative diseases that are not ‘primary’ tauopathies (tau pathology is, of course, a feature of AD, in combination with β-amyloid). Therefore, the new criteria do not identify primary tauopathies more accurately.

In order to assess whether individual clinical features were more commonly associated with CBD pathology, versus CBD mimics, we performed χ^2^ analysis on each feature. Several features were present more often in CBD mimic cases, including documented myoclonus (8/11 in CBD mimic cases compared with 2/15 in CBD pathology group, p=0.006) and visuospatial deficits (10/13 (77%) in CBD negative cases compared with 8/18 (44%) in CBD, p=0.15). However, no clinical features were significantly more common in the CBD group compared with CBD mimics.

## Discussion

The question for physicians and clinical trial investigators is simple: for a patient with clinically suspected CBD (or CBS) can we know whether they have CBD? The recent diagnostic criteria proposed by Armstrong *et al*^1^ are not sufficient to answer this question. The new criteria continue to misdiagnose CBD mimics as CBD, and fail to identify about a third of cases with CBD, even when applied late in the course of the disease.

We applaud the work of Armstrong *et al* in their aim of advancing a challenging area of clinical diagnostics and support the inclusion of a PSP syndrome and a FBS within the spectrum of CBS. These changes acknowledge the overlapping phenotypes of tau-related disorders, including PSP and FTLD-tau. However, our data demonstrate that the Armstrong criteria do not improve upon the longstanding difficulty of identifying patients with CBD sufficiently to improve the prospects of clinical trials. A third of patients with CBD do not meet the new criteria, while the new criteria fail to rule out common CBD mimics where patients with CBS have underlying non-CBD pathology.

We acknowledge potential shortcomings of this study, in particular those of sample size and retrospective analysis. The patient groups are relatively small compared with the data used to derive the Armstrong *et al* criteria. However, our study size is in the upper range of other published series of CBD and CBS, including those contributing to the Armstrong criteria, reflecting that CBD is a relatively uncommon disease.^1^ ^9^ ^10^ ^11^ Our data draw on detailed clinical phenotyping and pathological study over many years, and provide an objective evaluation of the new criteria in this context. A consequence of the retrospective nature of this study is that some clinical features might have been less well documented in some patients, and a unified clinical pro forma to ensure systematic recording of all features (present or absent) was only adopted in 2005. Omissions are, we suggest, more likely to reflect the absence of a particular feature in a given case, but this cannot be confirmed in retrospect. Prospectively collected data based on these criteria will be helpful in resolving some of these issues.

The chief difficulty of distinguishing CBD from CBD mimics lies in the very similar CBS phenotypes arising from CBD and non-CBD pathologies. Surprisingly, none of the individual clinical features are more prevalent with CBD than non-CBD cases of CBS, suggesting that analyses of clinical features alone are insufficient for accurate diagnosis. A solution may be to combine clinical with biomarker data to provide additional information, including neuroimaging biomarkers or cerebrospinal fluid (CSF) biochemistry. This approach was used by Burrell *et al*^12^ who used the positron emission tomography (PET) ligand PiB to study patients with clinical CBD (ie, CBS) to investigate differences in clinical phenotype between those with extensive amyloid burden and those without, albeit without neuropathology. Rabinovici and colleagues compared the sensitivity and specificity of flurodeoxyglucose-PET and PiB-PET in patients with AD and frontotemporal dementia. In neuropathologically confirmed cases, the authors found that PiB was slightly superior with a sensitivity of 89.5% for AD and specificity of 83%.^13^ However, PiB correlates poorly with the distribution of hypometabolism and clinical syndromes between different variants of AD.^14^ CSF biomarkers are also an area of active investigation in CBD. Borroni *et al*,^15^ for example, reported that CSF tau to Aβ ratio correlated with ^99m^Tc-ECD single photon emission CT scan results suggestive of a diagnosis of Alzheimer's pathology, although also without neuropathological confirmation of diagnosis. Validating such neuroimaging or CSF biomarker findings with neuropathology will be important in the absence of an accurate ‘gold standard’ by clinical diagnostic criteria. Our study lacks such biomarkers. It is possible that a clinical diagnosis of CBS together with negative biomarkers for AD pathology (eg, negative PiB/low CSF tau to Aβ ratio) would accurately identify cases of CBD, but this requires confirmation. The development of novel tau ligands for PET imaging^16^ ^17^ may also lead to an important role of PET in the accurate diagnosis of CBD and CBD mimics.

The difficulty of reliably detecting CBD pathology continues to slow the development and application of effective mechanism-based therapies. Higher accuracy would increase power to detect a therapeutic effect, compared with trials which include a substantial proportion of AD or TDP-43-based FTLD. Better criteria for the diagnosis of CBD and CBS are still required, which may include imaging biomarkers.
